# DDAH1/ADMA Regulates Adiponectin Resistance in Cerebral Ischemia via the ROS/FOXO1/APR1 Pathway

**DOI:** 10.1155/2022/2350857

**Published:** 2022-04-25

**Authors:** Yichen Zhao, Minjie Zhang, Yunxiao Dou, Kangshuai Du, Xueyuan Liu, Yanxin Zhao

**Affiliations:** Department of Neurology, Shanghai Tenth People's Hospital, Tongji University School of Medicine, No. 301 Middle Yanchang Road, Shanghai 200072, China

## Abstract

Dimethylarginine dimethylaminohydrolase 1 (DDAH1) protects against cerebral ischemia injury via regulating the level of asymmetric dimethylarginine (ADMA). This study is aimed at exploring the effect of adiponectin resistance on ADMA-induced neuronal loss in ischemic stroke (IS) and the underlying mechanism. DDAH1 knockout (DDAH1^−/−^) and wild-type (DDAH1^+/+^) rats underwent middle cerebral artery occlusion/reperfusion (MCAO/R). Plasma and brain adiponectin levels and the expressions of adiponectin receptor 1 (APR1), adaptor protein, phosphotyrosine interacting with PH domain and leucine zipper 1 (APPL1), adenosine monophosphate-activated protein kinase (AMPK), and phosphorylated AMPK were determined after 24 h, 3 days, and 7 days. Neurological behavior, infarct volume, and adiponectin signaling were evaluated using adiponectin peptide or AdipoRon. The levels of reactive oxygen species (ROS) and Forkhead box O1 (FOXO1) (a transcription factor for APR1) were also assessed. An oxygen-glucose deprivation/reoxygenation (OGD/R) model was established in primary neurons. DDAH1 was overexpressed in neurons, after which FOXO1 expression, ROS production, adiponectin resistance, and cell viability were detected. DDAH1^−/−^ rats showed no significant difference in adiponectin level in either plasma or brain after MCAO/R in DDAH1^+/+^ rats, but downregulated APR1 expression and suppressed adiponectin signaling were observed. AdipoRon, but not adiponectin peptide, attenuated the neurological deficits and adiponectin resistance in DDAH1^−/−^ rats. ROS accumulation and phosphorylated FOXO1 expression also increased with DDAH1 depletion. Following DDAH1 overexpression, decreased cell viability and inhibited adiponectin signaling induced by OGD/R were alleviated in primary neurons, accompanied by reduced ROS production and phosphorylated FOXO1 expression. Our study elucidated that in IS, DDAH1 protected against adiponectin resistance in IS via the ROS/FOXO1/APR1 pathway.

## 1. Introduction

Stroke is the primary cause of death and the dominant cause of disability worldwide, and ischemic stroke (IS) is the most common type of stroke [1]. Recently, the association between IS and adiponectin (APN) has attracted great interest among researchers. APN is one of the major adipocyte-derived hormones, and it exerts protective effects such as increased insulin sensitivity, antiatherosclerosis, and anti-inflammation by activating its receptors [2]. APN receptors are comprised of two subtypes, and adiponectin receptor 1 (APR1) is the main isoform. Most of the beneficial effects of APN are mediated by activation of the APR1/adaptor protein, phosphotyrosine interacting with PH domain and leucine zipper 1 (APPL1)/adenosine monophosphate-activated protein kinase (AMPK) pathway [3].

Evidence has suggested that APN and APR1 are widely expressed in the brain and play crucial roles in ischemic injury [4]. Although emerging data have indicated that adiponectin supplementation enhances brain tolerance against ischemia, plasma APN level before and after stroke or whether APN level can be used to predict stroke occurrence, and outcome was controversial. Clinical observations and experimental studies have suggested APR downregulation or phosphorylation under pathological conditions, named as “APN resistance,” which impairs APN signaling [5]. However, the mechanisms underlying APN resistance in IS remain unknown.

As a biomarker of endothelial dysfunction, asymmetric dimethylarginine (ADMA) has been identified as the risk factor for various cardiovascular conditions such as pulmonary hypertension, coronary heart disease, and portal hypertension [6–8]. Our previous study also revealed the protective role of dimethylarginine dimethylaminohydrolase 1 (DDAH1) (main hydrolase for ADMA) in IS and IS-induced blood–brain barrier disruption via downregulating ADMA [9]. The opposite effects of ADMA and APN in various clinical situations have been clarified [10, 11]. However, their relationship in the brain and the effect of ADMA on APN resistance are still unclear.

Forkhead box O1 (FOXO1), which belongs to the forkhead family of transcription factors, has been reported to positively mediate APR1 transcription by directly binding to the APR1 gene promoter region [12, 13]. The phosphorylation of FOXO1 (pFOXO1) and its subsequent translocation from the nucleus to the cytoplasm effectively reduce its transcriptional capabilities, which are sensitive to reactive oxygen species (ROS) [14]. A recent study has suggested the ROS/FOXO1/APR1 axis as a cause of adiponectin resistance in skeletal muscle [15]. Also, widespread concern exists over the association between ADMA and oxidative stress, especially ROS generation and its downstream signaling [16]. Therefore, the link between ADMA and ROS/FOXO1/APR1 axis is worth exploring.

Using the DDAH1 knockout rat strain, we explored the effect of APN resistance on ADMA-aggravated ischemic injury. The middle cerebral artery occlusion/reperfusion (MCAO/R) rat model was used to mimic IS pathogenesis in humans. The levels of plasma and brain APN and the expressions of APR, APPL1, and phosphorylated AMPK (pAMPK)/AMPK for 24 h, 3 days, and 7 days were assessed to ensure downregulated APN signaling with DDAH1 depletion. APN peptide (APNp) or AdipoRon administration was performed to further ascertain APN resistance, after which the neurological behavior, infarct volume, and APN resistance at 24 h were evaluated. ROS accumulation and FOXO1 expression were also analyzed. An oxygen-glucose deprivation/reoxygenation (OGD/R) model was also established in primary neurons, and the adiponectin signaling, FOXO1 expression, and cell viability were assessed. The present study is aimed at exploring the mechanisms underlying the protective effect of DDAH1 in IS. We hypothesized that DDAH1 alleviated APN resistance via ROS/FOXO1/APR1 pathway.

## 2. Materials and Methods

### 2.1. Animals

DDAH1 knockout (DDAH1^−/−^) Sprague–Dawley (SD) rats were kindly provided by Professor Da-Chun Xu (Department of Cardiology, Shanghai Tenth People's Hospital, Tongji University School of Medicine). The Clustered Regularly Interspaced Short Palindromic Repeats (CRISPR)/CRISPR-associated protein 9 (CRISPR/Cas9) technique was applied to generate DDAH1^−/−^ rats on the SD background as previously illustrated [17]. Wild-type male SD rats (DDAH1^+/+^), weighing 250–280 g, were purchased from Shanghai SIPPR-BK Laboratory Animal Co. Ltd. All rats were housed in separated cages in specific-pathogen-free animal rooms at 25°C with humidity of 40% on a 12 h light/dark cycle and were given free access to water and diet. In addition, all animal studies (including the rat euthanasia procedure) were conducted according to the guidelines recommended by the 1986 U.K. Animals (Scientific Procedures) Act.

### 2.2. MCAO/R Model

All rats were fasted but had free access to water 12 h before establishing the MCAO/R model. Each rat was weighed before the experiment and anesthetized through intraperitoneal injection with 2% pentobarbital sodium (0.2 mL/100 g). Rectal temperature was maintained at 36.5°C to 37.5°C. The rats were subjected to MCAO/R as previously described [9]. Briefly, following a midline incision, the neck vessels, including the left common carotid artery, internal carotid artery (ICA), and external carotid artery (ECA), were exposed and isolated. Then, a small incision was made on the ECA, and a silica suture (Doccol Co.403756PK5Re) was gently advanced a distance of 18–20 mm into the ICA for to occlude the origin of the middle cerebral artery. The suture was removed carefully to restore perfusion 90 min later. After the neck incision was closed, the rats were allowed the access to food and water. In the nonischemic group, the rats underwent the same procedure without inserting the suture. After surgery, the rats were individually housed and carefully fed until the end of the experiment.

### 2.3. Drug Administration

APNp (Sangon Biotech, China) was diluted with 0.9% saline solution to a concentration of 5 mg/mL and intraperitoneally injected at a dose of 20 *μ*g/(g body weight) immediately after artery occlusion. The rats in the AdipoRon group received a regular diet containing AdipoRon. AdipoRon (30 mg/kg, Sigma) was mixed into the standard chow diet for 4 weeks ahead of establishing the MCAO/R model.

### 2.4. Assessment of the Behavior Score and the Infarct Volume

The neurobehavioral changes in rats were evaluated using the neurological severity score (NSS) (adapted from [18]). It consisted of four parameters, including motor function, sensory function, reflex absence, and beam balance and abnormal movements, and the total score was 18 points (Table S).

To measure the infarct volume, we performed triphenyltetrazolium chloride (TTC) staining 24 h after the surgery. The rats were anesthetized, and the brains were then collected and quickly frozen at –20°C for 20 min. The brain tissue was sliced into six coronal sections (2 mm thick) using Leica blades in a rat brain matrix (RWD Life Science Co.). The slices were stained with TTC dye (Servicebio, Co.) at 37°C in the dark for 30 min and fixed with 4% paraformaldehyde (Servicebio, Co.). The infarcted tissue was unstained and white, while the stained normal tissue was red. The digital images of slices were analyzed using ImageJ software. The infarct area on each slice was corrected by the standard method (infarct area = contralateral hemisphere area − area of nonischemic ipsilateral hemisphere) to avoid possible interferences of brain edema. The infarct volume was calculated by multiplying the total infarct area with the thickness of each slice. The percentages of infarct volume were also determined by the infarct area/contralateral hemisphere area ratio.

### 2.5. Determination of Plasma APN Level

The plasma APN level was measured using an enzyme-linked immunosorbent assay (ELISA) kit (Abcam) following the manufacturer's protocols. Each sample was gauged in triplicate. A standard curve was generated, and the corresponding optical density (OD) values (450 nm) were measured to determine the concentration of the unknown sample.

### 2.6. Western Blot Analysis

The expression levels of APN, APR1, APPL1, AMPK, pAMPK, FOXO1, and pFOXO1 in the brain and primary neurons were analyzed using Western blot analysis. Proteins from the brain tissues around the infarct area and primary neurons were collected using a protein extraction kit (Epizyme). Bicinchoninic acid assay was used to detect the concentration of proteins. An equal number of proteins in different groups were separated by Tris–glycine sodium dodecyl sulfate-polyacrylamide gel electrophoresis and transferred to nitrocellulose membranes. Consequently, the membranes were blocked with 5% nonfat milk in PBST at room temperature for 1 h and incubated with primary antibodies (anti-APN, Abcam; anti-APR1, Proteintech; anti-APPL1, Proteintech; anti-AMPK, Abcam; anti-pAMPK, Abcam; anti-FOXO1A, Abcam; anti-pFOXO1A, Abcam; and anti-ACTB antibody, Sigma) at 4°C overnight. On the following day, the membranes were incubated with corresponding HRP-conjugated secondary antibody (Abcam) at room temperature for 1 h. The protein band was scanned on Amersham Imager 600 using an enhanced chemiluminescence kit (Sangon Biotech (Shanghai) Co.), and the relative amounts of proteins were analyzed using ImageJ software.

### 2.7. Reverse Transcription–Quantitative Polymerase Chain Reaction

The mRNA levels of APR1 and DDAH1 in the infarct area and primary neurons were analyzed using reverse transcription–quantitative polymerase chain reaction (RT-qPCR). The brain tissues (50 mg) around the infarct area were extracted to collect total RNA. The RNA concentration and purity were estimated using an ultraviolet spectrophotometer, and the OD260/OD280 ratio was determined to be between 1.8 and 2.0. The RNA was reverse transcribed into cDNA using a PrimeScript RT reagent kit (Takara Bio Inc., Otsu, Japan), and the obtained cDNA was stored at –20°C. The primers for glyceraldehyde-3-phosphate dehydrogenase (GAPDH), APR1, and DDAH1 of rats were designed and synthesized by Shanghai Sangon Biotechnology Co., Ltd. (Shanghai, China). The primer sequences used were as follows: APR1: 5′-ATATAAGGTCTGGGAGGGGC-3′ (forward), 5′-CCAGTCAGGAAGCACATCGT-3′ (reverse); DDAH1: 5′-CAGCCACCCCTCGGTCTT-3′ (forward), 5′-AAGCCGTCCACCTTTTCCAT-3′ (reverse); GAPDH: 5′-ACCACAGTCCATGCCATCAC-3′ (forward), 5′-TCCACCACCCTGTTGCTGTA-3′ (reverse). RT-qPCR was conducted with the cDNA template following the instructions of the SYBR FAST qPCR Master Mix kit (KAPA) by using an ABI7500 qPCR instrument (7900, Applied Biosystems, CA, USA), and GAPDH was used as an internal control. The 2^−ΔΔCt^ method was employed to calculate the expression level of APR1 mRNA. The experiment was repeated three times.

### 2.8. Detection of ROS Production

The fluorogenic dye 2′,7′-dichlorofluorescin-diacetate (DCFH-DA, Bestbio) that could be oxidized by ROS was used to detect the ROS level. After the rats were anesthetized and perfused, the brains were quickly collected and sliced into 15 *μ*m thick coronal sections. The sections were then mounted onto polylysine-coated slides and incubated in 10 *μ*M DCFH-DA for 30 min. The fluorescence was measured at excitation–emission spectra of 495–529 nm. To detect cellular ROS accumulation, the treated primary neurons were seeded in a 24-well plate and loaded with 10 *μ*M DCFH-DA and incubated at 37°C for 30 min. The plates were then washed, and the fluorescence was measured using a plate reader at 495 nm. The values were normalized to the control.

### 2.9. Isolation and Culture of Primary Neurons

The primary neurons were isolated from SPF E18 SD rats. After the pregnant rats were anesthetized, the uterus was exposed, and the embryos were removed. The embryonic brain was dissected and immediately placed in the ice vessel containing Dulbecco's modified Eagle's medium (Gibco). The cortex tissues were isolated under a dissecting microscope and digested using 0.125% trypsin at 37°C for 10 min. After separation and centrifugation, the obtained neurons were inoculated into six-well plates after the wells were treated with poly-D-lysine (Sigma–Aldrich) for 4–6 h. The cells were maintained using Neurobasal Medium (Gibco) supplemented with 1× B27 (Gibco) and 1× GlutaMAX (Gibco) at 37°C with 5% CO_2_. The neurons were cultured in deoxygenated, glucose-free Hanks' balanced salt solution at 37°C with 5% CO_2_ and 95% N_2_ for 24 h to establish the oxygen-glucose deprivation/reperfusion (OGD/R) model and then cultured under normoxic conditions. ADMA was reconstituted in distilled water, prior to each experiment. DDAH1-overexpressed (PGMLV-CMV-R_DDAH1-green fluorescent protein (GFP)) and negative control plasmids (GFP) were purchased from Genomeditech (Shanghai, China). The synthesized plasmids were transfected into the neurons using OptiMEM and Lipofectamine 2000 (Invitrogen, Thermo Fisher Scientific, USA) following the supplier's protocol. The overexpression efficiency of DDAH1 mRNA and protein was assessed using RT-qPCR and Western blot analyses.

### 2.10. Cell Viability Measurement

Cell viability was assessed by the cell counting kit-8 (CCK-8) method. Briefly, CCK-8 solution was added to the cells in 96-well plates. After incubation at 37°C for 1 h, the OD value was measured using an absorbance microplate reader at 450 nm. The cell viability was expressed as a percentage of the control culture OD value.

### 2.11. Determination of the ADMA Level

Plasma and tissue ADMA levels were measured using an ELISA kit (JianglaiBio, Shanghai, China) following the manufacturer's protocol. Each sample was gauged in triplicate. A standard curve was generated using six standard concentrations, and the corresponding OD values (450 nm) was measured to determine the concentration of the unknown sample.

### 2.12. Immunofluorescence Staining

Immunofluorescence staining was performed to explore the expression and distribution of pFOXO1 in the neurons. The seeded neurons were incubated with pFOXO1 primary antibodies (Proteintech) and corresponding secondary antibodies (Jackson Immuno Research). The cells were viewed and photographed at a magnification of ×600. The relative fluorescence intensity and the nuclear-to-cytosolic fluorescence ratio were analyzed using ImageJ software.

### 2.13. Statistical Analysis

All data were shown as mean ± standard error of mean (SEM) and analyzed using the SPSS version 22.0. Data sets were tested for equal variances using Levene's test. Differences among groups were analyzed using one-way or two-way analysis of variance (ANOVA) and Kruskal–Wallis ANOVA, and the least significance difference (LSD) test was used for post hoc comparisons. A *P* value < 0.05 indicated a statistically significant difference (^∗^*P* < 0.05, ^∗∗^*P* < 0.01, ^∗∗∗^*P* < 0.001).

## 3. Results

### 3.1. Similar Plasma and Brain APN Levels between DDAH1^−/−^ and DDAH1^+/+^ Rats after Establishing MCAO/R Model

**Figure 1:**
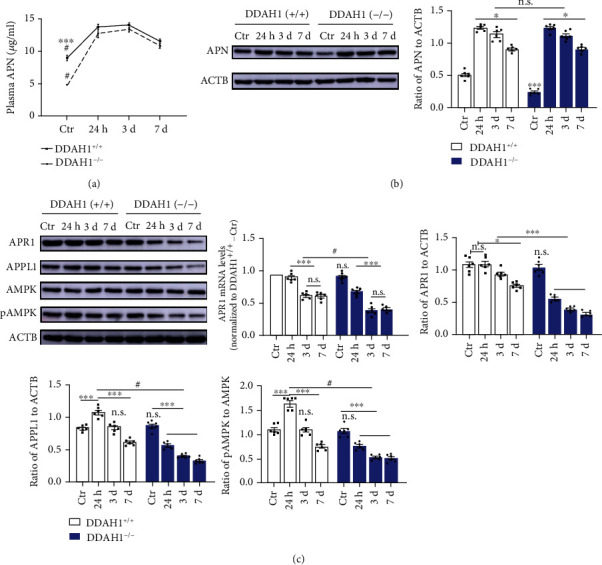
DDAH1 depletion aggravated the ischemia-induced APN resistance. (a) Statistic analysis of plasma APN levels in normal condition and at 24 h, 3 day, and 7 day after MCAO/R model. ^∗∗∗^*P* < 0.001 versus DDAH1^−/−^ rats, ^#^*P* < 0.001 versus 24 h, 3 day, and 7 day. (b) Representative Western blots of APN around infarct area and statistical analysis and quantification of APN expression. ^∗∗∗^*P* < 0.001 versus DDAH1^+/+^ rats, n.s.: *P* > 0.05, ^∗^*P* < 0.05. (c) Representative Western blots of APR1, APPL1, AMPK, and pAMPK around infarct area and statistical analysis and quantification. n.s.: *P* > 0.05 versus DDAH1^+/+^ rats, ^#^*P* < 0.001 versus DDAH1^+/+^ rats, ^∗∗∗^*P* < 0.001, ^∗^*P* < 0.05. Data are presented as mean ± SEM. *n* = 6. APN: adiponectin; DDAH1: dimethylarginine dimethylamino hydrolase 1; Ctr: control; APR1: adiponectin receptor 1; APPL1: adaptor protein, phosphotyrosine interacting with PH domain and leucine zipper 1; AMPK: adenosine monophosphate-activated protein kinase; pAMPK: phosphorylated AMPK.

We previously found increased plasma and brain ADMA levels under both normal and ischemic conditions in DDAH1^−/−^ rats with aggravated neurological damage after establishing the MCAO/R model. Hence, we explored the APN levels in DDAH1^−/−^ rats. Under normal conditions, the APN levels in the plasma and brain were downregulated after DDAH1 depletion compared with that in DDAH1^+/+^ rats. After establishing the MCAO/R model, the APN levels in the plasma and brain infarct area significantly increased in both DDAH1^−/−^ and DDAH1^+/+^ rats, reaching a peak after 24 h and then gradually declined over the next 7 days. Interestingly, although the APN baseline level was significantly lower after DDAH1 depletion, no significant difference was found under DDAH1 depletion condition following the establishment of the MCAO/R model (Figures 1(a) and 1(b)). This suggested that DDAH1/ADMA might not affect the process of cerebral ischemia purely via regulating APN level.

### 3.2. Aggravated Ischemic Damage and Earlier Occurrence of APN Resistance in DDAH1^−/−^ Rats after Establishing the MCAO/R Model

We further examined the APR1 expression level in infarct tissue and investigated the possible role of APN signaling in ADMA-aggravated ischemic damage. The mRNA and protein expression levels of APR1 were not significantly altered after DDAH1 depletion under the nonischemic conditions (Figure 1(c)). The expression level of APR1 was basically unchanged in DDAH1^+/+^ rats 24 h after MCAO/R, began to decline 3 days later, and remained constant till day 7. Nevertheless, the APR expression level was significantly decreased after 24 h in DDAH1^−/−^ rats compared with the baseline and remained relatively low over the next 7 days (Figure 1(c)).

As the key components of APN signaling, the expressions of APPL1, AMPK, and pAMPK were also detected (Figure 1(c)). Consistent with APR expression, the expression of APPL1 and the pAMPK/AMPK ratio were not significantly changed after DDAH1 depletion under normal conditions, while both of them decreased 24 h after MCAO/R. However, the level of APPL1 and the pAMPK/AMPK ratio in DDAH1^+/+^ rats were significantly increased after 24 h and gradually decreased over the next 7 days following MCAO/R. The aforementioned findings indicated aggravated APN resistance in cerebral ischemia after DDAH1 depletion.

### 3.3. AdipoRon, but Not APNp, Restored the Neurological Function and Attenuated the APN Resistance in DDAH1^−/−^ Rats after Establishing the MCAO/R Model

**Figure 2:**
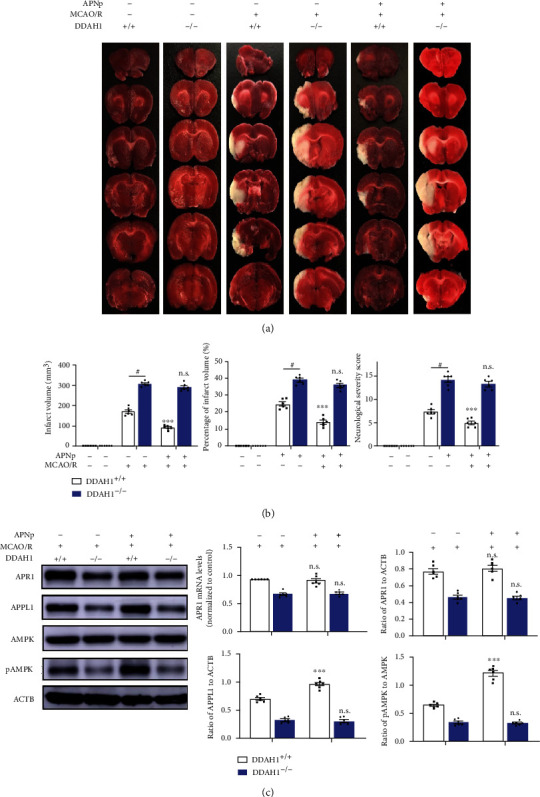
APN peptide did not restore the APN signaling in DDAH1^−/−^ rats after MCAO/R model. (a) Representative 24-hour TTC staining of rat brain sections. The white-colored areas represented infarct region in these sections, and red-colored areas represented normal region. (b) Statistical analysis of NSS, infarct volume, and its percentage. n.s.: *P* > 0.05 versus without APNp injection, ^∗∗∗^*P* < 0.001 versus without APNp injection, ^#^*P* < 0.001. (c) Representative Western blots of APR1, APPL1, AMPK, and pAMPK around infarct area and statistical analysis and quantification. n.s.: *P* > 0.05 versus without APNp injection, ^∗∗∗^*P* < 0.001 versus without APNp injection. Data are presented as mean ± SEM. *n* = 6. APNp: adiponectin peptide; DDAH1: dimethylarginine dimethylamino hydrolase 1; MCAO/R: middle cerebral artery occlusion/reperfusion; APR1: adiponectin receptor 1; APPL1: adaptor protein, phosphotyrosine interacting with PH domain and leucine zipper 1; AMPK: adenosine monophosphate-activated protein kinase; pAMPK: phosphorylated AMPK.

We evaluated the alterations of the neurological deficit with APNp administration to ensure the APN resistance of DDAH1^−/−^ rats. In DDAH1^+/+^ rats, APNp treatment significantly reduced the NSS and infarct volume after 24 h. However, the NSS and infarct volume of DDAH1^−/−^ rats were slightly decreased via APNp, with no significant difference (Figures 2(a) and 2(b)). The expressions of APR1 and APPL1, AMPK, and pAMPK were also detected. APNp did not significantly influence the APR1 expression in DDAH1^+/+^ rats, but it significantly upregulated the expression of APPL1 and pAMPK/AMPK ratio 24 h after MCAO/R. In the DDAH1^−/−^ rats, no significant difference was found in terms of the expressions of APR1, APPL1, or pAMPK/AMPK ratio via APNp supplementation (Figure 2(c)).

**Figure 3:**
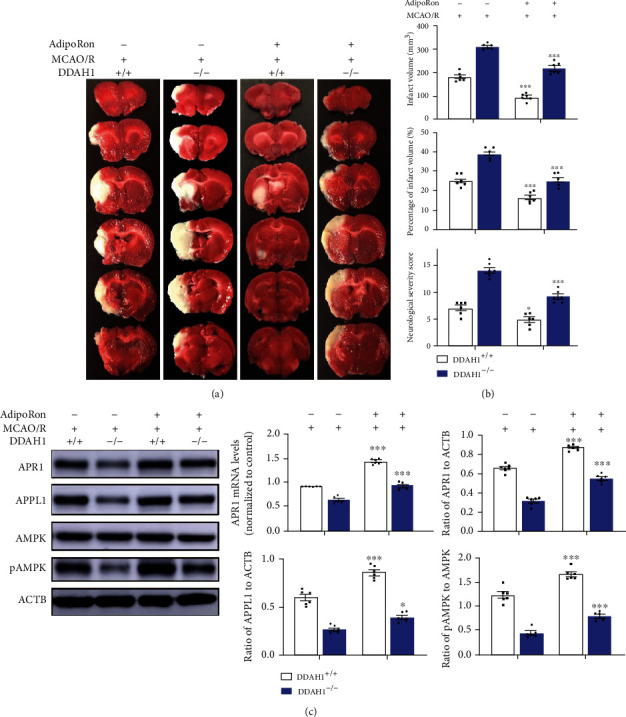
AdipoRon alleviated the APN resistance in DDAH1^−/−^ rats after MCAO/R model. (a) Representative 24-hour TTC staining of rat brain sections. The white-colored areas represented infarct region in these sections, and red-colored areas represented normal region. (b) Statistical analysis of NSS, infarct volume, and its percentage. ^∗∗∗^*P* < 0.001 versus without AdipoRon treatment; ^∗^*P* < 0.05 versus without AdipoRon treatment. (c) Representative Western blots of APR1, APPL1, AMPK, and pAMPK around infarct area and statistical analysis and quantification. ^∗∗∗^*P* < 0.001 versus without AdipoRon treatment, ^∗^*P* < 0.05 versus without AdipoRon treatment. Data are presented as mean ± SEM. *n* = 6. DDAH1: dimethylarginine dimethylamino hydrolase 1; MCAO/R: middle cerebral artery occlusion/reperfusion; APR1: adiponectin receptor 1; APPL1: adaptor protein, phosphotyrosine interacting with PH domain and leucine zipper 1; AMPK: adenosine monophosphate-activated protein kinase; pAMPK: phosphorylated AMPK.

The alterations in neurological deficit after AdipoRon treatment were measured to determine the effect of APR on ADMA-aggravated ischemic injury. Following AdipoRon diet, the neurological damage after 24 h was alleviated in DDAH1^+/+^ rats, exhibiting significantly decreasing NSS and infarct volume. In addition, AdipoRon treatment also significantly reduced 24 h NSS and infarct volume in DDAH1^−/−^ rats (Figures 3(a) and 3(b)). The effect of AdipoRon on APN signaling was subsequently assessed. APR1 expressions in DDAH1^−/−^ and DDAH1^+/+^ rats were significantly upregulated. AdipoRon also significantly enhanced the expression of APPL1 and pAMPK/AMPK ratio after MCAO/R in both DDAH1^−/−^ and DDAH1^+/+^ rats (Figure 3(c)).

### 3.4. Enhanced ROS Accumulation and pFOXO1 Expression in DDAH1^−/−^ Rats after Establishing the MCAO/R Model

**Figure 4:**
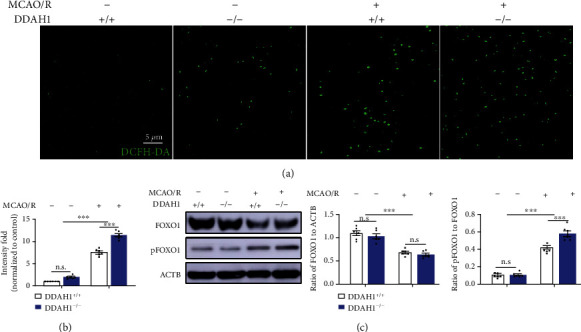
DDAH1 depletion enhanced the ischemia-induced ROS accumulation and pFOXO1 upregulation. (a) Representative images of DCFH-DA staining for ROS measurement. (b) Statistical analysis of ROS fluorescence intensity. n.s.: *P* > 0.05, ^∗∗∗^*P* < 0.001. (c) Representative Western blots of FOXO1 and pFOXO1 around infarct area and statistical analysis and quantification. n.s.: *P* > 0.05, ^∗∗∗^*P* < 0.001. Data are presented as mean ± SEM. *n* = 6. DDAH1: dimethylarginine dimethylamino hydrolase 1; MCAO/R: middle cerebral artery occlusion/reperfusion; DCFH-DA: 2′,7′-dichlorofluorescin-diacetate; FOXO1: forkhead box O1; pFOXO1: phosphorylated FOXO1.

We then detected the ROS level and FOXO1 expression in the infarct area to explore the mechanisms underlying reduced APR1 expression. A slight increase in the ROS level was found in DDAH1^−/−^ rats compared with DDAH1^+/+^ rats under nonischemic conditions, with no significant difference (Figures 4(a) and 4(b)). Following MCAO/R, a significant increase in relative fluorescence was observed compared to the normal conditions; DDAH1 depletion further increased ROS accumulation (Figures 4(a) and 4(b)). Significant downregulation of FOXO1 expression was found in both DDAH1^−/−^ and DDAH1^+/+^ rats following MCAO/R, with no significant difference. pFOXO1 level was then assessed. No significant difference in the pFOXO1/FOXO1 ratio was found under nonischemic conditions. Interestingly, the pFOXO1/FOXO1 ratio was significantly increased in both DDAH1^−/−^ and DDAH1^+/+^ rats following MCAO/R, while it was significantly higher in DDAH1^−/−^ rats (Figure 4(c)).

### 3.5. DDAH1 Overexpression Attenuated OGD/R-Induced Cell Death via the ROS/FOXO1/APR1 Pathway

**Figure 5:**
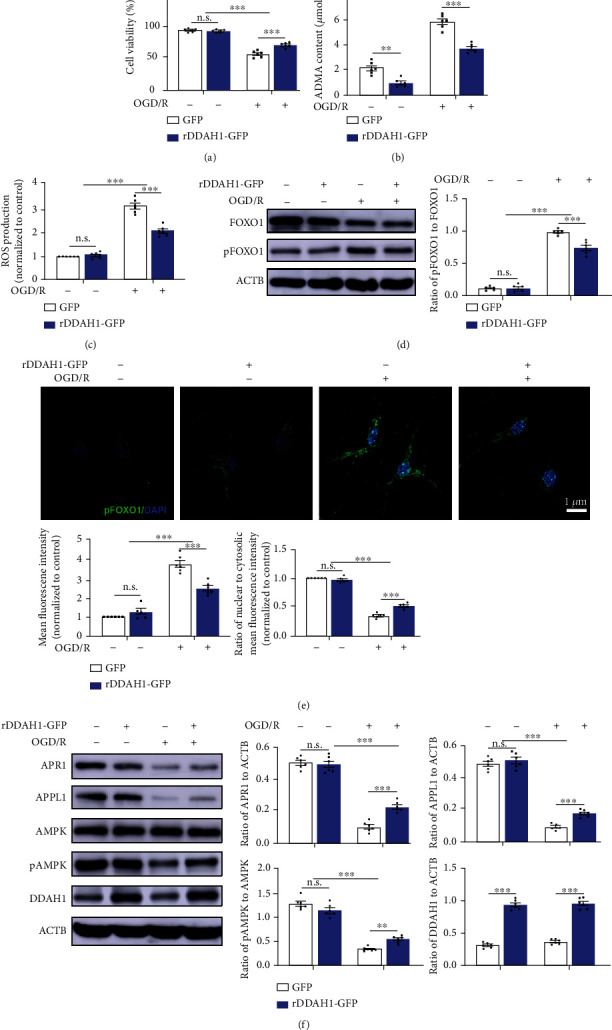
DDAH1 overexpression attenuated OGD/R-induced cell death via the ROS/FOXO1/APR1 pathway. (a–c) Quantifications of the cell viability, ADMA concentrations, and ROS levels within primary neurons. n.s.: *P* > 0.05, ^∗∗^*P* < 0.01, ^∗∗∗^*P* < 0.001. (d) Representative Western blots of FOXO1 and pFOXO1 in primary neurons and statistical analysis and quantification. n.s.: *P* > 0.05, ^∗∗∗^*P* < 0.001. (e) Representative images of immunofluorescence staining for pFOXO1 and statistical analysis for nuclear-to-cytosolic fluorescence ratio of pFOXO1. n.s.: *P* > 0.05, ^∗∗∗^*P* < 0.001. (f) Representative western blots of APR1, APPL1, AMPK, pAMPK, and DDAH1 and statistical analysis and quantification. n.s.: *P* > 0.05, ^∗∗^*P* < 0.01, ^∗∗∗^*P* < 0.001. Data are presented as mean ± SEM. *n* = 6. ADMA: asymmetric dimethylarginine; ROS: reactive oxygen species; OGD/R: oxygen-glucose deprivation/reoxygenation; DDAH1: dimethylarginine dimethylamino hydrolase 1; GFP: green fluorescent protein; FOXO1: forkhead box O1; pFOXO1: phosphorylated FOXO1; APR1: adiponectin receptor 1; APPL1: adaptor protein, phosphotyrosine interacting with PH domain and leucine zipper 1; AMPK: adenosine monophosphate-activated protein kinase; pAMPK: phosphorylated AMPK.

An *in vitro* study was also performed to further investigate the effect of DDAH1 on the ROS/FOXO1/APR1 pathway in the background of DDAH1 overexpression. Cell viability of primary neurons was significantly reduced in the OGD/R model. Following DDAH1 overexpression, the mRNA and protein levels of DDAH1 were upregulated, and the OGD/R-induced cell death was alleviated in primary neurons (Figure S1a-b and Figure 5(a)). Cellular ADMA concentration was found to increase significantly following OGD/R, while ADMA level was decreased under both normal and OGD/R conditions after overexpressing DDAH1 (Figure 5(b)).

OGD/R led to increased ROS production in neurons, while DDAH1 overexpression significantly decreased the ROS level (Figure 5(c)). FOXO1 expression was reduced after OGD/R in both GFP and rDDAH1-GFP groups, with no significant difference. The pFOXO1/FOXO1 ratio was significantly upregulated in the OGD/R model, but the ratio was decreased following DDAH1 overexpression (Figure 5(d)). Consistent with the result of western blot analysis, neurons receiving OGD/R treatment exhibited a significant increased fluorescence of pFOXO1, which was reversed by DDAH1 overexpression. In terms of localization, the mean fluorescence intensity of pFOXO1 in nuclear and cytosolic areas was also measured separately. The nuclear-to-cytosolic pFOXO1 fluorescence ratio was reduced following OGD/R and was significantly increased after overexpressing DDAH1 (Figure 5(e)).

The effects of OGD/R and DDAH1 overexpression on APN signaling were also examined. In the OGD/R model, primary neurons showed downregulated expressions of APR1 and APPL1 and a decreased pAMPK/AMPK ratio. DDAH1 overexpression alone had no effect on APR1/APPL1/pAMPK signaling, while it reversed the inhibition of APR1/APPL1/pAMPK signaling induced by OGD/R in neurons (Figure 5(f)).

ADMA treatment was performed to further confirm the regulatory role of DDAH1. The cells were treated with proportional series concentrations of ADMA (0, 20, 40, 80, and 160 *μ*M) for 24 h. ADMA (>40 *μ*M) exhibited significant cytotoxicity (*P* < 0.001) in a concentration-dependent manner. Cell activity was also assessed at various time points in the presence of 40 *μ*M ADMA, and significant cytotoxicity was found after 24 h (Figure S1c). The effect of ADMA on DDAH1-GFP-transfected neurons was first explored under normal condition. After ADMA treatment, ADMA level was significantly increased in the neurons transfected with both GFP and DDAH1-GFP (Figure S2b). Although exposure to ADMA decreased the cell viability, DDAH1 overexpression attenuated the cytotoxicity of ADMA (Figure S2a). ROS production was slightly increased after ADMA treatment, which was not significant (Figure S2c). There was also no significant change in the expression of FOXO1 and pFOXO1/FOXO1 ratio or the nuclear-to-cytosolic pFOXO1 fluorescence ratio after ADMA treatment (Figure S2d-e).

**Figure 6:**
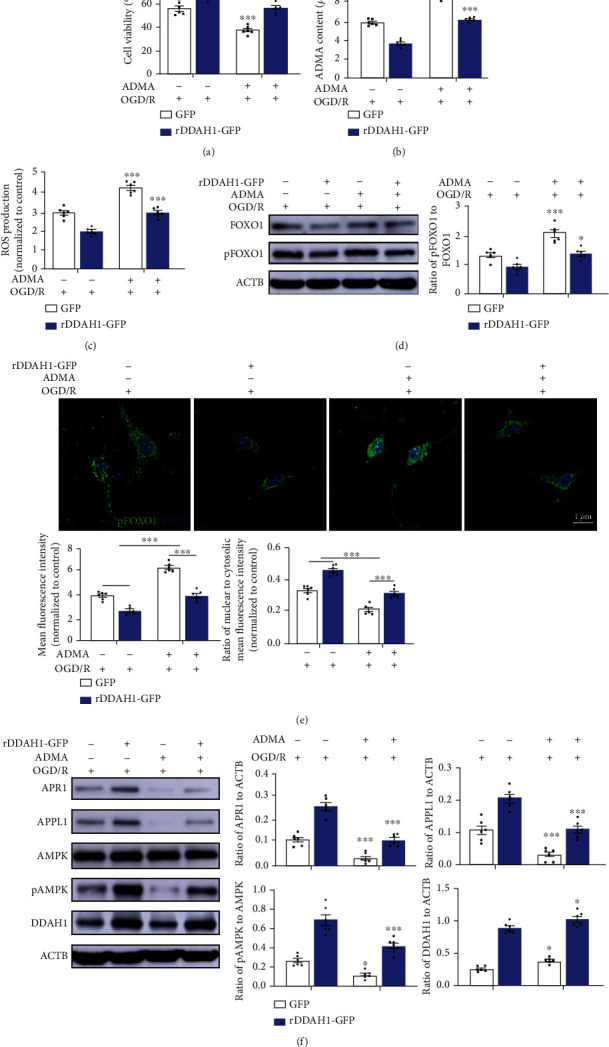
ADMA diminished the protective effect of DDAH1 on OGD/R-induced cell death. (a–c) Quantifications of the cell viability, ADMA concentrations, and ROS levels within primary neurons. ^∗∗^*P* < 0.01 versus without ADMA treatment, ^∗∗∗^*P* < 0.001 versus without ADMA treatment. (d) Representative Western blots of FOXO1 and pFOXO1 in primary neurons and statistical analysis and quantification. n.s.: *P* > 0.05 versus without ADMA treatment, ^∗^*P* < 0.05 versus without ADMA treatment, ^∗∗∗^*P* < 0.001 versus without ADMA treatment. (e) Representative images of immunofluorescence staining for pFOXO1 and statistical analysis for nuclear-to-cytosolic fluorescence ratio of pFOXO1. ^∗∗∗^*P* < 0.001. (f) Representative Western blots of APR1, APPL1, AMPK, pAMPK, and DDAH1 and statistical analysis and quantification. n.s.: *P* > 0.05 versus without ADMA treatment, ^∗^*P* < 0.05 versus without ADMA treatment, ^∗∗^*P* < 0.01 versus without ADMA treatment, ^∗∗∗^*P* < 0.001 versus without ADMA treatment. Data are presented as mean ± SEM. *n* = 6. ADMA: asymmetric dimethylarginine; ROS: reactive oxygen species; OGD/R: oxygen-glucose deprivation/reoxygenation; DDAH1: dimethylarginine dimethylamino hydrolase 1; GFP: green fluorescent protein; FOXO1: forkhead box O1; pFOXO1: phosphorylated FOXO1; APR1: adiponectin receptor 1; APPL1: adaptor protein, phosphotyrosine interacting with PH domain and leucine zipper 1; AMPK: adenosine monophosphate-activated protein kinase; pAMPK: phosphorylated AMPK.

The effect of ADMA on DDAH1-GFP-transfected neurons was also examined in the OGD/R model. ADMA supplementation aggravated the neuronal death induced by OGD/R and also alleviated the protective effect of DDAH1 on cell viability (Figure 6(a)). In addition, increased ADMA concentration in neurons of the OGD/R model and diminished effect of DDAH1 on ROS accumulation were observed (Figures 6(b) and 6(c)). The pFOXO1/FOXO1 ratio, especially cytosolic pFOXO1 level, was also upregulated by ADMA treatment following DDAH1 overexpression (Figures 6(d) and 6(e)). Furthermore, ADMA supplementation exacerbated the decline in neuronal APN signaling following OGD/R (Figure 6(f)). The regulatory effect of DDAH1 on APR1/APPL1/pAMPK signaling was also reversed by ADMA treatment.

## 4. Discussion

The association between IS and APN resistance has recently been recognized. However, its underlying mechanism remains unclear. Our previous study showed that DDAH1 played a protective role in IS via regulating ADMA levels. Opposite effects of ADMA and APN in cardiovascular diseases have been widely recognized. However, the link between ADMA and APN resistance, especially in IS pathology, is still unclear. This study investigated the effect of DDAH1 on APN resistance in IS and explored the underlying mechanism.

Adiponectin is secreted by adipocytes and regulating metabolic processes and insulin sensitivity. It is thought to play anti-inflammatory, antioxidant, and antiatherogenic roles. Several studies have shown that serum adiponectin levels are inversely correlated with the incidence and severity of metabolic dysfunction and cardiovascular diseases, such as obesity, diabetes mellitus, coronary heart disease, and hypertension [19–22]. In the brain, adiponectin also plays a role in neuroprotection in various disease states including stroke [23]. Several studies have indicated adiponectin-mediated protective effects against stroke. APN-knockout mice exhibited increased cerebral infarct volume after ischemia and reperfusion, while exogenous adiponectin reduced the infarct volume in both APN-knockout and wild-type mice [24, 25]. In the meantime, adiponectin overexpression increased positive behavioral outcomes and stimulated angiogenesis following cerebral ischemic injury [26].

ADMA was considered as the marker of endothelial dysfunction and a cardiovascular risk factor for various diseases. Our previous study found that DDAH1 depletion in rats showed aggravated neurological behavioral outcomes and increased infarct volume after establishing the MCAO/R model at 24 h, 3 day, and 7 day time points, which were accompanied by an upregulated ADMA level. Nevertheless, the mechanisms underlying ADMA impairment in IS pathology are yet to be fully clarified. Several studies have demonstrated a negative correlation between ADMA and APN [27–29]. However, their association in the central nervous system diseases, especially stroke, needs further exploration. In this study, we examined plasma and brain APN levels in DDAH1^−/−^ rats under both normal and ischemic conditions. DDAH1^−/−^ rats showed lower plasma and brain APN levels under nonischemic conditions. Following MCAO/R, plasma APN level and APN expression in the ischemic hemisphere of both DDAH1^−/−^ and DDAH1^+/+^ rats were significantly increased, reaching a peak after 3 days, and decreased thereafter. Interestingly, no significant differences were found between DDAH1^−/−^ and DDAH1^+/+^ rats. This suggested that DDAH1/ADMA might not contribute to the pathogenesis of cerebral ischemia purely through modulating APN levels.

Peripheral APN crosses the blood–brain barrier and acts through its two receptors APR1 and APR2. Further, APR1 and APR2 both are expressed in mammalian cortical neurons, with APR1 being more pronounced than APR2 [30, 31]. The neuroprotective effect of APN was found to be mediated via the APR1/APPL1/AMPK signaling pathway [2]. APPL1 acts as an immediate downstream effector of APR1 to mediate APN-induced phosphorylation of AMPK, which plays a protective role in brain damage after ischemia [32, 33]. Emerging experimental and clinical evidence have indicated that besides hypoadiponectinemia, reduced biologic response to APN (APN resistance) exists under many pathological conditions. The underlying mechanisms of APN resistance involves the downregulation or phosphorylation of APR [5]. In the present study, we detected the APR1 expression and APN signaling in the ischemic hemisphere 24 h, 3 days, and 7 days after MCAO/R. After 24 h, APR1 expression was slightly increased (but not significantly) compared with that under normal conditions. During the next few days, it gradually decreased, which suggested APN resistance during ischemic injury. No significant difference was observed between DDAH1^−/−^ and DDAH1^+/+^ rats under normal conditions, while APR1 expression in DDAH1^−/−^ rats was significantly decreased after 24 h and also decreased over the next 7 days. The mRNA expression of APR1 in DDAH1^−/−^ and DDAH1^+/+^ rats exhibited the same trend, suggesting that the downregulation of APR1 was aggravated following DDAH1 depletion. APN downstream signaling was found to be enhanced after 24 h in DDAH1^+/+^ rats and then was gradually alleviated after 3 days and 7 days. However, the APN signaling began to decrease after 24 h in DDAH1^−/−^ rats, and this decreasing trend was significantly higher. APN resistance in ischemic injury seemed to exacerbate after DDAH1 depletion.

Exogenous APN and AdipoRon were administered to further explore the regulatory effect of DDAH1/ADMA on APN resistance. Pretreatment with recombinant human APN or APNp could confer neuroprotection against cerebral ischemia–reperfusion injury [34–36]. AdipoRon is a small-molecule agonist of APR, which ameliorates insulin resistance via the APR1/AMPK pathway [37]. It was also considered to protect against myocardial ischemia–reperfusion injury, cognitive dysfunction in Alzheimer's disease, and secondary brain injury after intracerebral hemorrhage [38–40]. In our study, APNp treatment significantly improved the neurological behavioral outcome and decreased the infarct volume in DDAH1^+/+^ rats with enhanced APPL1/pAMPK signaling, while the expression of APR1 was not significantly changed. However, APNp administration did not alleviate the neurological damages in DDAH1^−/−^ rats nor changed the APR1/APPL1/pAMPK signaling. However, AdipoRon pretreatment attenuated the cerebral ischemia–induced injury in both DDAH1^−/−^ and DDAH1^+/+^ rats and also enhanced the APR1/APPL1/pAMPK signaling. The finding that AdipoRon, but not APNp, diminished the effects of DDAH1 depletion indicates that DDAH1/ADMA may exert its role in IS pathology via regulating APN resistance.

The expression of APR1 has been shown to be stimulated by a transcription factor named FOXO1 [41]. Upon phosphorylation, FOXO1 relocates from the nucleus to the cytoplasm, which effectively reduces its transcriptional capabilities. FOXO1 phosphorylation was reported to be enhanced by excessive ROS production [15]. Meanwhile, it was widely accepted that ROS was closely linked to ADMA [42, 43]. Thus, we next detected FOXO1 expression and ROS level to determine whether ADMA regulated the FOXO1 expression by promoting ROS production. Under nonischemic conditions, the relative ROS intensity in DDAH1^−/−^ rats was slightly increased, which was not significant. Following MCAO/R, enhanced ROS accumulation was exhibited in DDAH1^−/−^ rats compared with DDAH1^+/+^ rats. FOXO1 expression was significantly decreased after MCAO/R, with no significant difference between DDAH1^−/−^ and DDAH1^+/+^ rats. Nevertheless, the expression of pFOXO1/FOXO1 was increased after MCAO/R, and DDAH1^−/−^ rats showed a significantly higher pFOXO1/FOXO1 ratio compared with DDAH1^+/+^ rats.

We also transfected rDDAH1-GFP plasmid into primary neurons to overexpress DDAH1. The results showed that DDAH1 overexpression restored cell viability after OGD/R injury, which was associated with decreased ADMA and ROS levels. Despite no significant change in FOXO1 expression, the pFOXO1/FOXO1 ratio was significantly decreased following DDAH1 overexpression. Meanwhile, the upregulation of cytosolic pFOXO1 level was shown to be inhibited in the context of DDAH1 overexpression. As expected, APR1/APPL1/pAMPK signaling axis was also enhanced and restored. The addition of ADMA slightly reduced the cell viability under normal condition, but it did not significantly influence the ROS accumulation or pFOXO1/FOXO1 ratio. However, in neurons with OGD/R model, the treatment of ADMA diminished the protective effects of DDAH1 and further damaged the ROS/FOXO1/APR1 pathway.

## 5. Conclusions

In summary, APN resistance appeared in IS pathogenesis. This study firstly demonstrated that DDAH1 exerted its protective effect in IS via alleviating APN resistance. Our findings also suggested that this effect occurred via diminishing ADMA-induced oxidative stress and decreasing the expression of phosphorylated FOXO1, consequently increasing APR1 expression. Hence, DDAH1-induced restoration of APN resistance may provide new potential targets for the treatment of IS patients.

## Data Availability

The experimental data that are used to support the findings of this study are available from the corresponding author upon request.

## Supplementary Materials

Supplementary Materials Table S: neurological severity score assessment for neurobehavioral changes. Figure S1: (a) the semiquantitative analysis of DDAH1 mRNA levels. (b) Representative immunoblot of protein of DDAH1 in cells and the semiquantitative analysis of DDAH1 expression. (c) Quantifications of the cytotoxicity of ADMA in different concentrations and quantifications of the cytotoxicity of 40 *μ*M ADMA on primary neurons at various time points. The data are presented as mean ± SEM, *n* = 5 or 6. n.s.: *P* > 0.05, ^∗∗∗^*P* < 0.001. DDAH1: dimethylarginine dimethylamino hydrolase 1; GFP: green fluorescent protein; Ctr: control; ADMA: asymmetric dimethylarginine. Figure S2: (a–c) quantifications of the cell viability, ADMA concentrations, and ROS levels within primary neurons. n.s.: *P* > 0.05, ^∗^*P* < 0.05, ^∗∗∗^*P* < 0.001. (d) Representative western blots of FOXO1, pFOXO1, and DDAH1 in primary neurons and statistical analysis and quantification. n.s.: *P* > 0.05, ^∗∗∗^*P* < 0.001. (e) Representative images of immunofluorescence staining for pFOXO1 and statistical analysis for nuclear-to-cytosolic fluorescence ratio of pFOXO1. n.s.: *P* > 0.05. Data are presented as mean ± SEM. *n* = 6. ADMA: asymmetric dimethylarginine; ROS: reactive oxygen species; OGD/R: oxygen-glucose deprivation/reoxygenation; DDAH1: dimethylarginine dimethylamino hydrolase 1; GFP: green fluorescent protein; FOXO1: forkhead box O1; pFOXO1: phosphorylated FOXO1.
