# Supplementary material for: DDAH1/ADMA Regulates Adiponectin Resistance in Cerebral Ischemia via the ROS/FOXO1/APR1 Pathway
Source: Oxid Med Cell Longev. 2022 Apr 25;2022:2350857. doi: 10.1155/2022/2350857 (PMC9060971; doi:10.1155/2022/2350857)
Supplement: Supplementary Materials — Table S: neurological severity score assessment for neurobehavioral changes. Figure S1: (a) the semiquantitative analysis of DDAH1 mRNA levels. (b) Representative immunoblot of protein of DDAH1 in cells and the semiquantitative analysis of DDAH1 expression. (c) Quantifications of the cytotoxicity of ADMA in different concentrations and quantifications of the cytotoxicity of 40 μM ADMA on primary neurons at various time points. The data are presented as mean ± SEM, n = 5 or 6. n.s.: P > 0.05, ∗∗∗P < 0.001. DDAH1: dimethylarginine dimethylamino hydrolase 1; GFP: green fluorescent protein; Ctr: control; ADMA: asymmetric dimethylarginine. Figure S2: (a–c) quantifications of the cell viability, ADMA concentrations, and ROS levels within primary neurons. n.s.: P > 0.05, ∗P < 0.05, ∗∗∗P < 0.001. (d) Representative western blots of FOXO1, pFOXO1, and DDAH1 in primary neurons and statistical analysis and quantification. n.s.: P > 0.05, ∗∗∗P < 0.001. (e) Representative images of immunofluorescence staining for pFOXO1 and statistical analysis for nuclear-to-cytosolic fluorescence ratio of pFOXO1. n.s.: P > 0.05. Data are presented as mean ± SEM. n = 6. ADMA: asymmetric dimethylarginine; ROS: reactive oxygen species; OGD/R: oxygen-glucose deprivation/reoxygenation; DDAH1: dimethylarginine dimethylamino hydrolase 1; GFP: green fluorescent protein; FOXO1: forkhead box O1; pFOXO1: phosphorylated FOXO1. [file 2350857.f1.doc]

**Table S. Neurological severity score**

| **Motor tests** | **Score** |
| --- | --- |
| **Raising rat by tail(normal=0; maximum=3)** | **（3）** |
| Flexion of forelimb | 1 |
| Flexion of hindlimb | 1 |
| Head moved >10° to vertical axis within 30s | 1 |
| **Placing rat on floor** **(normal=0; maximum=3)** | **（3）** |
| Normal walk | 0 |
| Inability to walk straight | 1 |
| Circling toward paretic side | 2 |
| Falls down to paretic side | 3 |
| **Sensory tests(normal=0; maximum=2)** | **（2）** |
| Placing test (visual and tactile test) | 1 |
| Proprioceptive test (deep sensation, pushing paw against table edge to stimulate limb muscles) | 1 |
| **Beam balance tests** **(normal=0; maximum=6)** | **（6）** |
| Balances with steady posture | 0 |
| Grasps side of beam | 1 |
| Hugs beam and 1 limb falls down from beam | 2 |
| Hugs beam and 2 limbs fall down from beam, or spins on beam (>60s) | 3 |
| Attempts to balance on beam but falls off (>40s) | 4 |
| Attempts to balance on beam but falls off (>20s) | 5 |
| Falls off; no attempt to balance or hang on to beam (<20s) | 6 |
| **Reflex absence and abnormal movements** | **（4）** |
| Pinna reflex (head shake when auditory meatus is touched) | 1 |
| Corneal reflex (eye blink when cornea is lightly touched with cotton) | 1 |
| Startle reflex (motor response to a brief noise from snapping a clipboard paper) | 1 |
| Seizures, myoclonus, myodystony | 1 |
| **Maximum points** | **（18）** |


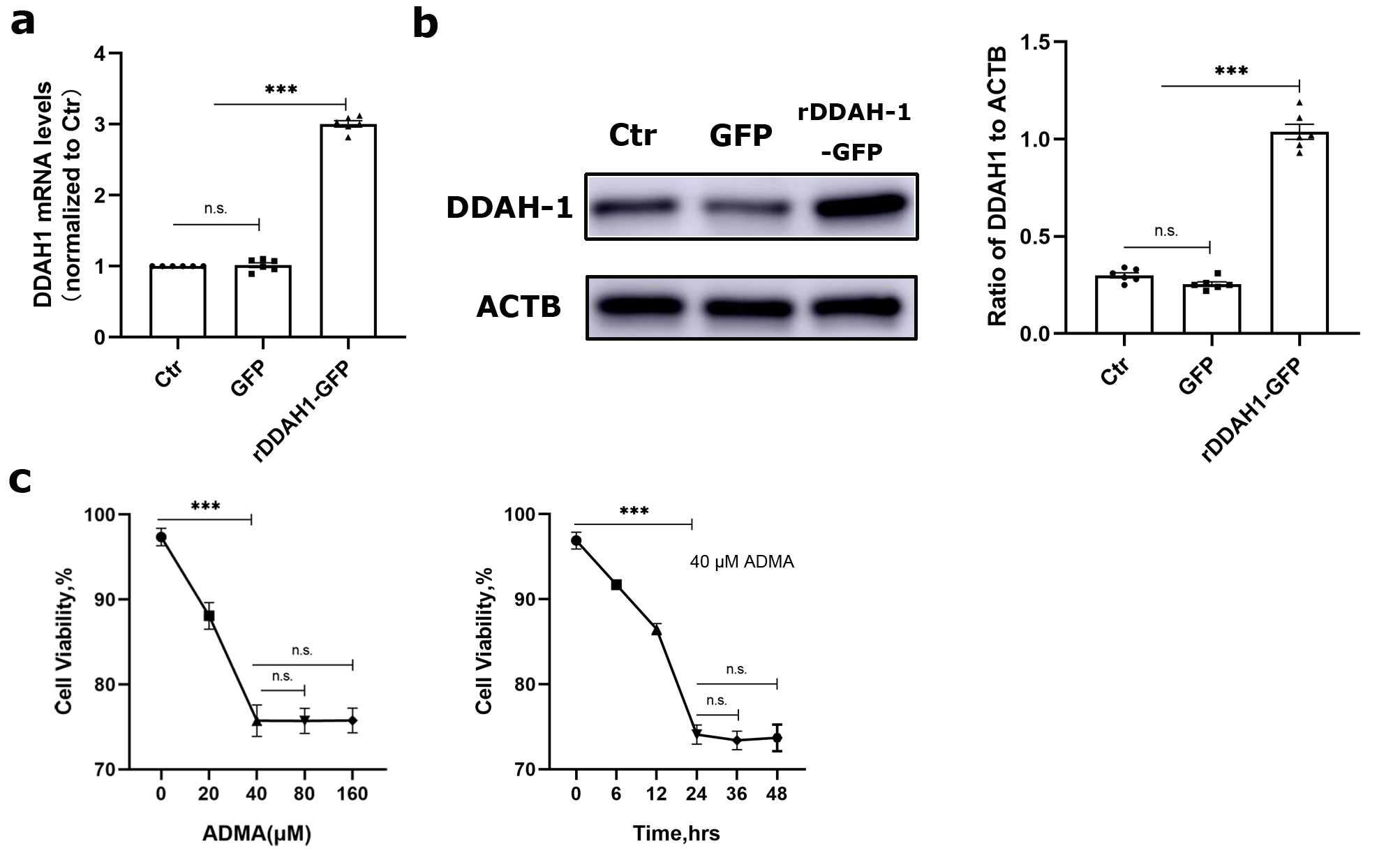


**Figure S1**

**
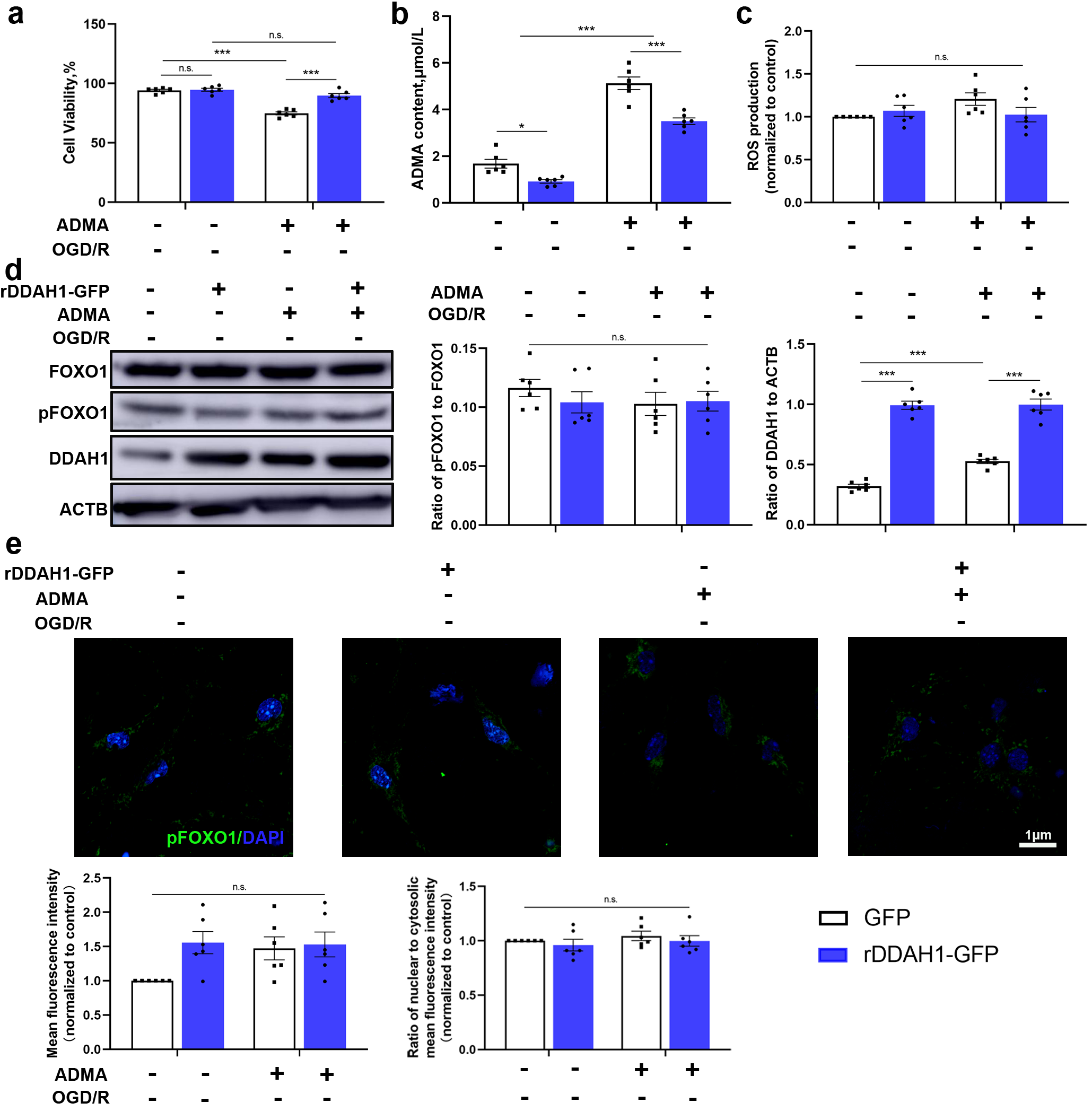
**

**Figure S2**
